# Unsupervised brain imaging 3D anomaly detection and segmentation with transformers

**DOI:** 10.1016/j.media.2022.102475

**Published:** 2022-07

**Authors:** Walter H.L. Pinaya, Petru-Daniel Tudosiu, Robert Gray, Geraint Rees, Parashkev Nachev, Sebastien Ourselin, M. Jorge Cardoso

**Affiliations:** a Department of Biomedical Engineering, School of Biomedical Engineering & Imaging Sciences, King's College London, London, UK; b UCL Queen Square Institute of Neurology, University College London, London, UK; c UCL Faculty of Life Sciences, University College London, London, UK

**Keywords:** Transformer, Unsupervised anomaly segmentation, Anomaly detection, Vector quantized variational autoencoder

## Abstract

Pathological brain appearances may be so heterogeneous as to be intelligible only as anomalies, defined by their deviation from normality rather than any specific set of pathological features. Amongst the hardest tasks in medical imaging, detecting such anomalies requires models of the normal brain that combine compactness with the expressivity of the complex, long-range interactions that characterise its structural organisation. These are requirements transformers have arguably greater potential to satisfy than other current candidate architectures, but their application has been inhibited by their demands on data and computational resources. Here we combine the latent representation of vector quantised variational autoencoders with an ensemble of autoregressive transformers to enable unsupervised anomaly detection and segmentation defined by deviation from healthy brain imaging data, achievable at low computational cost, within relative modest data regimes. We compare our method to current state-of-the-art approaches across a series of experiments with 2D and 3D data involving synthetic and real pathological lesions. On real lesions, we train our models on 15,000 radiologically normal participants from UK Biobank and evaluate performance on four different brain MR datasets with small vessel disease, demyelinating lesions, and tumours. We demonstrate superior anomaly detection performance both image-wise and pixel/voxel-wise, achievable without post-processing. These results draw attention to the potential of transformers in this most challenging of imaging tasks.

## Highlights

• We develop transformers for unsupervised brain imaging anomaly detection.
• Our approach combines a VQ-VAE with an ensemble of autoregressive transformers.
• Transformers outperform variational autoencoders across diverse classes of anomaly.
• Models are trained on UK Biobank MRI data, in 2D and 3D implementations.
• Evaluation includes white matter hyperintensities, tumours, and multiple sclerosis lesions.

## Graphical Abstract

**Figure:**
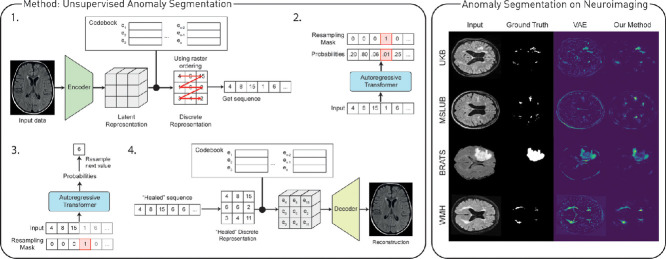

## 1 Introduction

Transformers have revolutionised language modelling, becoming the de-facto network architecture for language tasks (Radford et al., 2019, 2018; Vaswani et al., 2017). They rely on attention mechanisms to capture the sequential nature of an input sequence, dispensing with recurrence and convolutions entirely. This mechanism allows the modelling of dependencies of the inputs without regard to their distance, enabling the acquisition of complex long-range relationships. Since the approach generalises to any sequentially organised data, applications in other areas such as computer vision are increasingly seen, with impressive results in image classification (Chen et al., 2020; Dosovitskiy et al., 2020) and image synthesis (Child et al., 2019; Esser et al., 2020; Jun et al., 2020; Ramesh et al., 2021; Yu et al., 2021). The power to absorb relationships varying widely in their distance makes transformers of potential value in the arguably the hardest of neuroimaging tasks: anomaly detection.

The detection and segmentation of lesions in neuroimaging support an array of clinical tasks, including diagnosis, prognosis, treatment selection and mechanistic inference. However, the fine characterisation of these lesions requires an accurate segmentation which is generally both ill-defined and dependent on human expertise (Kamnitsas et al., 2017). Manual segmentation is expensive and time-consuming to obtain, greatly limiting clinical application, and the scale and inclusivity of available labelled data. Qualitative, informal descriptions or reduced measurements are often used instead in clinical routine (Porz et al., 2014; Yuh et al., 2012). For this reason, the development of accurate computer-aided automatic segmentation methods has become a major endeavour in medical image research (Menze et al., 2014). Most methods, however, depend on an explicitly defined target class, and are sensitive to the scale and quality of available labelled data, a sensitivity amplified by the many sources of complex variability encountered in clinical neuroimaging. Under real-world distributional shift, such models behave unpredictably, limiting clinical utility.

In recent years, many machine learning algorithms have been proposed for automatic anomaly detection. To overcome the necessity of expensive labelled data, unsupervised methods have emerged as promising tools to detect arbitrary pathologies (Baur et al., 2020b, 2018; Chen et al., 2020; Pawlowski et al., 2018), relying mainly on deep generative models of normal data to derive a probability density estimate of the input data defined by the landscape of normality. Pathological features then register as deviations from normality, avoiding the necessity for either labels or anomalous examples in training. The state of the art is currently held by variational autoencoder (VAE)-based methods (Baur et al., 2020a) which try to reconstruct a test image as the nearest sample on the learnt normal manifold, using the reconstruction error to quantify the degree and spatial distribution of any anomaly. This approach's success is limited by the fidelity of reconstructions from most VAE architectures (Dumoulin et al., 2016), and unwanted reconstructions of pathological features not present in the training data, suggesting a failure of the model to internalise complex relationships between remote imaging features.

In an effort to address these problems, we propose a method for unsupervised anomaly detection and segmentation using transformers, where we learn the distribution of brain imaging data with an ensemble of Performers (Choromanski et al., 2020). This study extends the details about the experiments that we performed on Pinaya et al. (2021), where we create and evaluate a robust method and compare its performance on synthetic and real datasets with recent state-of-the-art unsupervised methods. Besides that, we evaluate the performance of our method on 3D brain data for anomaly segmentation and detection.

### 1.1 Related work

Most previous unsupervised approaches can be categorized as reconstruction-based methods. These methods use models capable of outputting a “healed” version of the input data and relying on the pixel-wise residuals obtained from the difference to identify anomalies and lesions. Previously, these methods have used autoencoders (AE) (Baur et al., 2018; Chen and Konukoglu, 2018; Zimmerer et al., 2018), VAEs (Baur et al., 2018; Zimmerer et al., 2019), generative adversarial networks (Schlegl et al., 2019), and vector quantized variational autoencoders (VQ-VAE) (Marimont and Tarroni, 2021; Wang et al., 2020).

The closest studies to our own are the ones that rely on the VQ-VAE coupled with an autoregressive model with self-attention to help to create the healed version of the data. Wang et al. (2020) propose using a VQ-VAE to compress the input image and then obtain the probability distribution of this latent code using a PixelSNAIL (Chen et al., 2018). At the prediction stage, if the latent code is out-of-distribution, they use the PixelSNAIL to resample it. Similarly, Marimont and Tarroni (2021) use a PixelSNAIL, but they obtain multiple restorations by changing the temperature of the sampling operation and then weight them based on it. However, recent computer vision studies have pointed to the superiority in modelling the probability density estimate of the data using autoregressive transformers compared to the PixelSNAIL (Esser et al., 2020; Jun et al., 2020). We believe that a precise estimation of the likelihood of the latent variables is essential to determine which values need to be resampled to obtain a higher quality reconstruction.

Most of these previous studies rely only on the premise, common to reconstruction-based methods, that the raw pixel differences between the source and its reconstruction indicate the degree of anomaly. This introduces dependence on the fidelity and quality of the reconstructions, potentially resulting in residual maps that lack sufficient specificity. Here, we propose an alternative approach where the probabilities obtained from the transformer are used to identify the spatial characteristics of anomalous regions (more details in Section 2.4), decreasing dependence on the quality of the reconstructions of the underlying autoencoder.

## 2 Proposed method

The core of the proposed anomaly detector is a highly expressive transformer that learns the probability density function of healthy brain data. This requires us to express the contents of each image as a sequence of observations on which transformers-like models can operate. Owing to the size and complexity of brain imaging data, instead of learning the distributions on individual pixels or voxels directly, we use the compact latent discrete representation of a vector quantised variational autoencoder (Razavi et al., 2019; Van Den Oord et al., 2017). This approach allows us to compress the input data into a spatially smaller quantised latent representation, thus reducing the computational requirements and sequence length, making transformers feasible in neuroimaging applications.

### 2.1 Vector quantized variational autoencoder

**Fig. 1:**
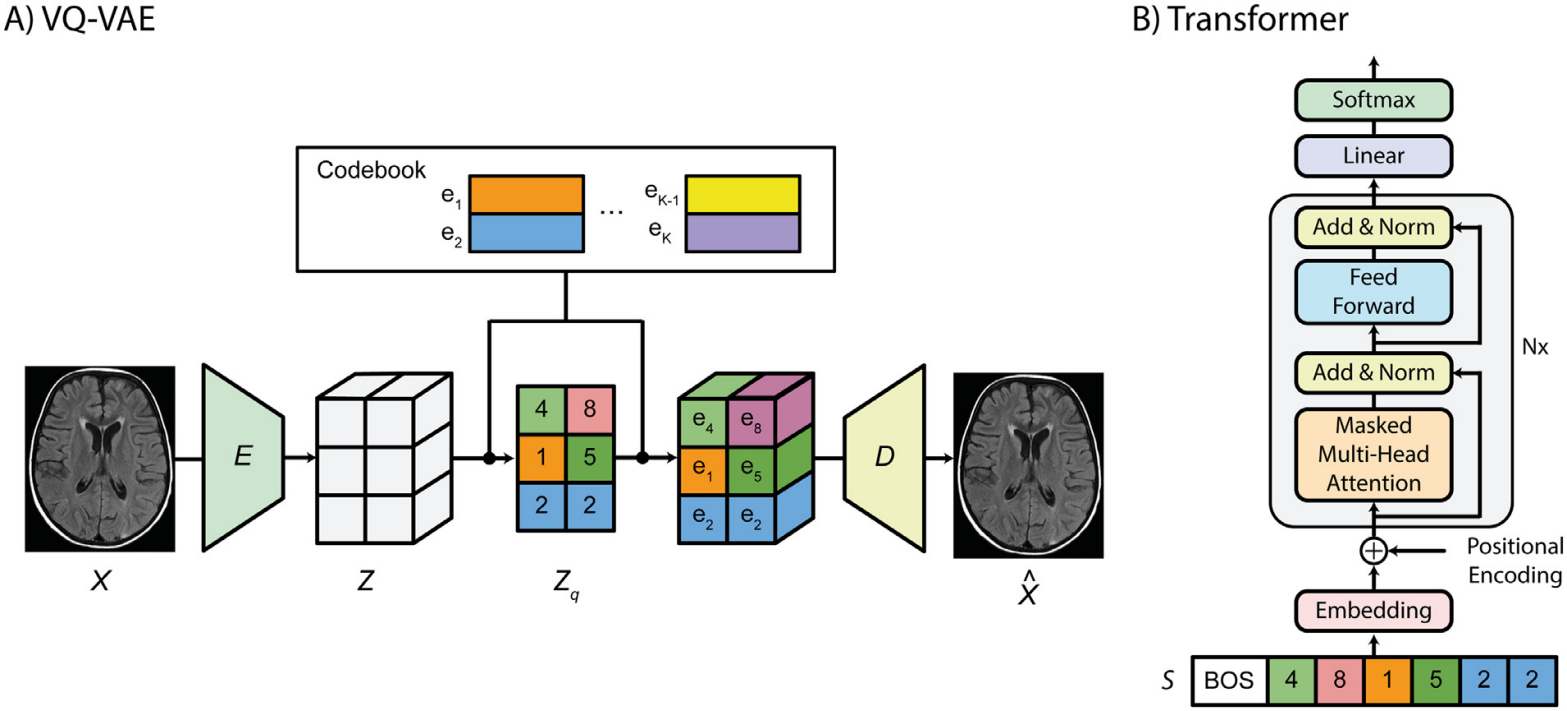
Our method uses a VQ-VAE to learn the latent discrete representation of brain data. This latent representation is transformed into a 1D sequence that is learned by the autoregressive transformer.

In the first step, we trained our VQ-VAE model. The VQ-VAE (Razavi et al., 2019; Van Den Oord et al., 2017) is a model that learns latent discrete representations of images (Fig. 1a). It comprises an encoder E that maps observations $x\in R^{D}$ onto a latent embedding space $z\in R^{d\times n_{z}}$, where $n_{z}$ is the dimensionality of each latent embedding vector, D and d are the spatial dimensions of the observations and latent embedding, respectively. An element-wise quantization is performed for each spatial code $z_{e}\in R^{n_{z}}$ onto its nearest vector $e_{k}\in R^{n_{z}}$, k∈1...K from a codebook, where K denotes the vocabulary size of the codebook and k is selected using $k=argmin_{j}{∥z_{e}-e_{j}∥}_{2}^{2}$. This codebook is learnt jointly with the other model parameters. A decoder G reconstructs the observations $\hat{x}\in R^{D}$ from the quantized latent space. We obtain the latent discrete representation $z_{q}\in R^{d}$ by replacing each code by its index k from the codebook.

In our training, the VQ-VAE loss function is described using the following:

(1) $$L_{VQVAE}=\;L_{recons}+L_{codebook}+\beta L_{commit}$$

(2) $$L_{recons}={∥x-\hat{x}∥}_{2}^{2}$$

(3) $$L_{codebook}={∥sg\left[z_{e}\right]-e_{k}∥}_{2}^{2}$$

(4) $$L_{commit}={∥sg\left[e_{k}\right]-z_{e}∥}_{2}^{2}$$

where the operator sg denotes the stop-gradient operation, which passes zero gradients during backpropagation. Here, the $L_{recons}$ penalizes for the distance between the input and its reconstruction, $L_{codebook}$ penalizes the codebook for the distance between the encodings $z_{e}$ and their nearest neighbours $e_{k}$ from the codebook, and $L_{commit}$ prevents the encodings from fluctuating too much, where β controls the reluctance to change the code corresponding to the encoder output. To speed up training, we used the exponential moving average updates for the codebook (Van Den Oord et al., 2017), as a replacement for the codebook loss.

### 2.2 Transformers

In the next step, we need an approach that explicitly models the likelihood function of the discrete elements from the latent representations. We chose to use autoregressive models, employing a transformer-based approach because transformers have consistently outperformed other autoregressive models (such as PixelCNN and PixelSNAIL) (Esser et al., 2020; Jun et al., 2020). After training the VQ-VAE, we assumed an arbitrary ordering to transform the latent discrete variables of healthy brains $z_{q}$ into a 1D sequence s and learn its probability density function p(s) using an autoregressive transformer (Fig. 1b).

During transformer training, given indices $s_{<i}$, the transformer learns to predict the distribution of the next indices $p\left(s_{i}\right)=\;p\left(s_{i}|s_{<i}\right)$. This way, we can compute the likelihood of the full sequence s as $p\left(s\right)=\;\prod_{i}p\left(s_{i}|s_{<i}\right)$ and we can maximize the training data's log-likelihood using the following loss function:

(5) $$L_{Transformer}=E_{x∼p\left(x\right)}\left[-\text{log}p\left(s\right)\right]$$

Since the transformer's attention mechanism relies on the inner products between all elements in the sequence, its computational costs scale quadratically with the sequence length. Several “efficient transformers” have recently been proposed to reduce this computational requirement (Tay et al., 2020). Our study uses the Performer, a model which uses an efficient (linear) generalized attention framework implemented by the FAVOR+ algorithm (Choromanski et al., 2020). This framework provides a scalable estimation of attention mechanisms expressed by random feature map decompositions, making transformers feasible for longer sequences, of the size needed for neuroimaging data.

### 2.3 Anomaly segmentation

**Fig. 2:**
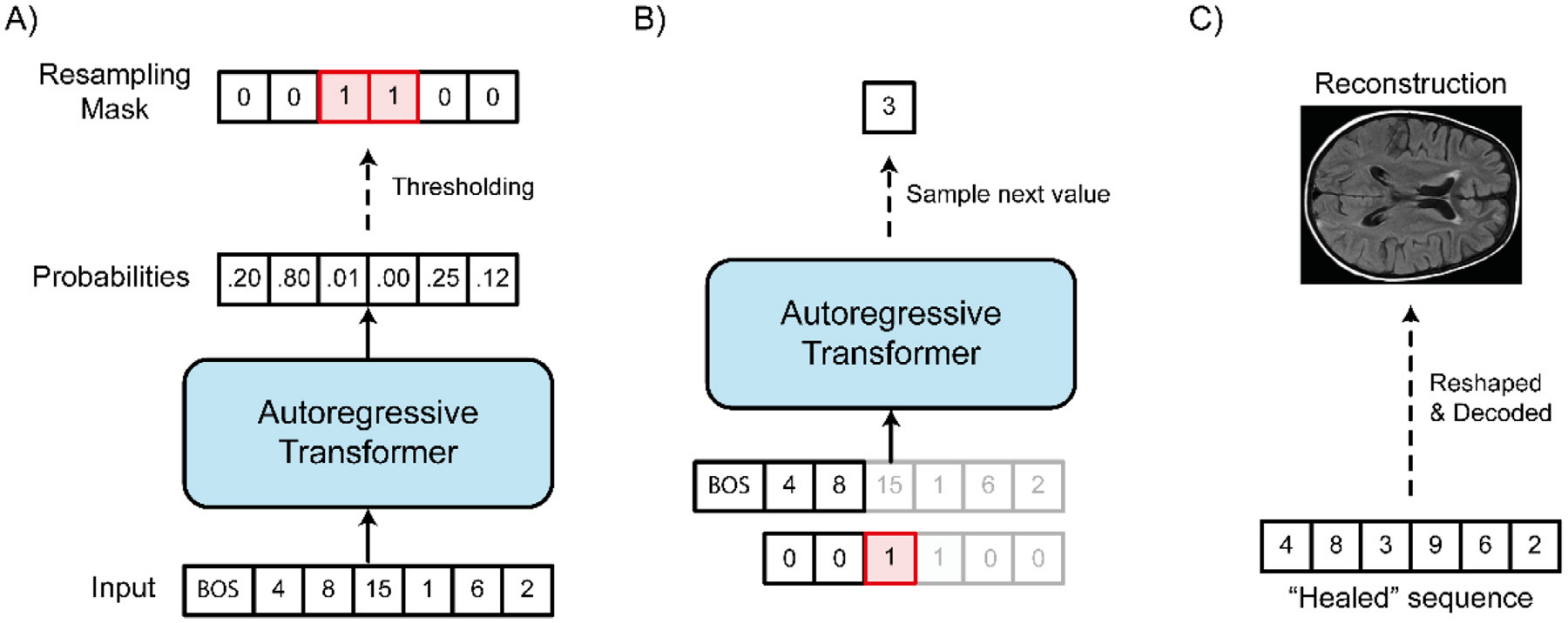
Anomaly segmentation method. A) the sequence obtained from the VQ-VAE is fed to the transformer with an “begin of sentence” token prepended. For each position of the sequence, the transformer will predict the value of the next element. Using the output probability of each real value, we apply a threshold (in this example, we use a threshold of 0.05) to identify which one is anomalous. A binary mask (the “resampling mask”) is created to indicate which value is below the threshold and should be corrected. B) For each position indicated in the resampling mask, we use the transformer to obtain values that have a higher probability of occurrence and we create a healed sequence. C) The healed 1-dimensional sequence is reshaped and processed by the VQ-VAE decoder to create a reconstruction without anomalies.

To segment an anomaly, first, we obtain the latent discrete representation $z_{q}$ from the VQ-VAE model. Next, we reshape $z_{q}$ into a sequence s, and we use the autoregressive transformer to obtain the likelihood of each latent variable value $p(s_{i})$ (Fig. 2a). These likelihood values indicate which latent variable has a low probability of occurring in normal data. Using an arbitrary threshold (we empirically determined a threshold of 0.005 on a holdout set for the 2D experiments and a threshold of 0.001 for the 3D experiments), we then can select indices with the lowest likelihood values and create a “resampling mask” m∈{0,1} where $m_{i}=\left\{1,\;\mathrm{if}\;p\left(s_{i}\right)\leq \mathrm{thre}\mathrm{shold};\;0,\;\mathrm{othe}\mathrm{rwise}\right\}$. The resampling mask indicates which latent variables are abnormal and should be corrected to produce a “healed” version of the sequence $\hat{s}$. For every position i from $\hat{s}$, if $m_{i}=0$, then ${\hat{s}}_{i}=s_{i}$; if $m_{i}=1$, then ${\hat{s}}_{i}∼p\left({\hat{s}}_{i}\;|{\hat{s}}_{<i}\right)$. This way, we replace the abnormal values with values sampled by the transformer (Fig. 2b). After we obtain $\hat{s}$, we use the inverse ordering operation and reshape our 1-dimensional sequence back to the original $z_{q}$ shape (i.e., d). This discrete latent representation is then decoded by G to obtain the reconstruction ${\hat{x}}^{\prime }\;$without the anomalies, in “healed” form (Fig. 2c). Finally, we obtain the pixel-wise residuals from the difference $|x-{\hat{x}}^{\prime }|$. The anomalies are segmented by thresholding the highest residuals values.

### 2.4 Spatial information from the resampling mask

Most previous anomaly detectors based on autoencoders are highly dependent on the reconstructive fidelity of the autoencoder. However, autoencoders (in special, variational autoencoders) are known for creating blurry reconstructions at reasonable compression rates (Dumoulin et al., 2016). These blurry reconstructions can create residual maps with high values in areas of the image with fine details, creating false positives and reducing the method specificity. Since our method relies on VQ-VAE to obtain ${\hat{x}}^{\prime }$, it is susceptible to the same problem. To mitigate the influence of blurry reconstructions, we exploit spatial information present in the “resampling mask”.

**Fig. 3:**
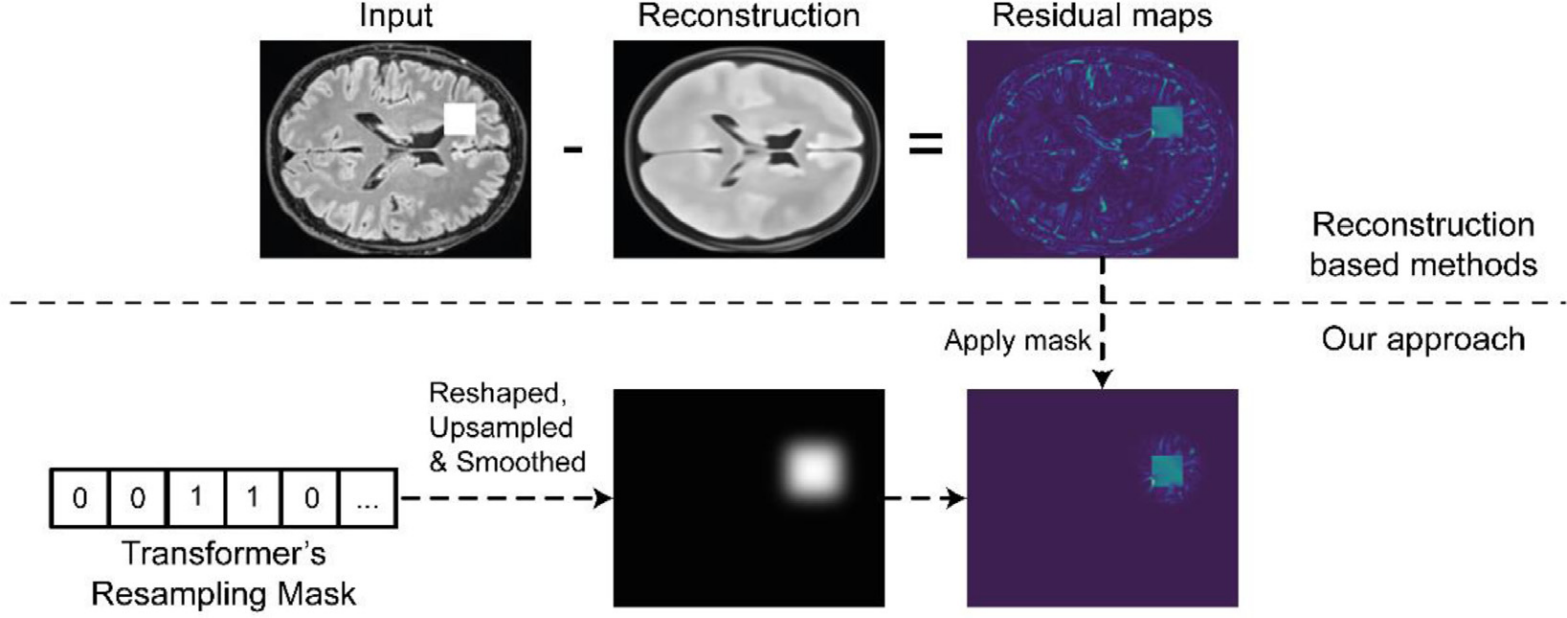
Using the spatial information from the resampling mask to improve segmentation. First, we reshape the resampling mask back to the format of the VQ-VAE latent space. Then, we upsample it to have the input image shape and we smooth it with a Gaussian filter. Finally, we use this mask to filter the residuals maps obtained from the difference between the inputted image and its healed reconstruction.

The resampling mask m indicates the position in the sequence that, according to the transformer, has a low likelihood of occurrence in the dataset with healthy brain data. If we use the same inverse ordering and reshape operations that we applied to $\hat{s}$, we obtain a 2D (or 3D) coarse-grained information about the location of the anomalies in the input space (again, according to the transformer). Since our VQ-VAE is relatively shallow, this latent space mask preserves most of the spatial information of the input data. As expected, it lacks precision when delineating the contours of the anomalies, but we can still use this spatial information from the latent space to avoid mislabelling finely detailed regions. This is achieved by upscaling the reshaped resampling mask from the latent space resolution to the input data resolution. Next, we smooth the mask using a Gaussian filter, and finally, we multiply the mask with the residuals (Fig. 3). This approach “cleans” areas of the residuals that were not specified as anomalous by our transformer but where the reconstructions might be largely due to lack of VQ-VAE capacity.

### 2.5 Multiple views of the latent space through reordering

The proposed resampling mask is not only used to select latent variables to "heal" the abnormal regions, but also to mask noisy residuals. The accuracy of our anomaly segmentation therefore heavily depends on its quality. To maximise it, we employ state-of-the-art autoregressive models based on transformers (Esser et al., 2020; Jun et al., 2020; Ramesh et al., 2021; Yu et al., 2021). Besides that, inspired by Choi et al. (2018), we also made our method more robust using an ensemble of models. Using the same VQ-VAE model, we trained an ensemble of autoregressive transformers. However, unlike Choi et al. (2018), each of our transformers uses a different reordering of the latent representation to create a sequence.

**Fig. 4:**
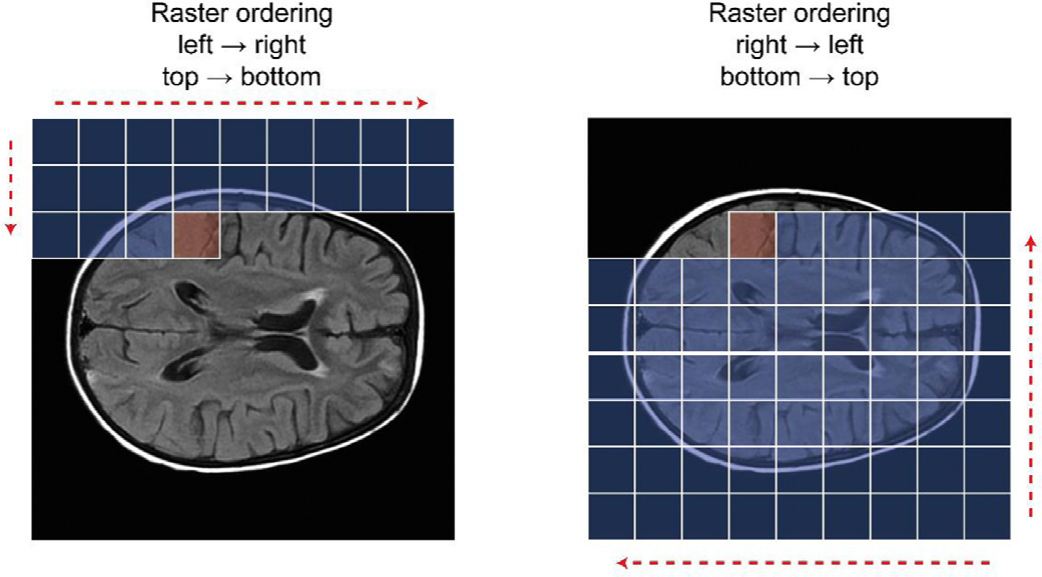
To predict the probability of the value in the red square, the transformer using the ordering of the left image (raster ordering, left → right, top → bottom) mostly uses the information of the image background as context (blue squares). If the transformer uses the ordering of the right image (raster ordering, right → left, bottom → top), it will have a richer context, with more information about the brain, that could help make a more accurate prediction about the value in the red square.

The autoregressive nature of transformers means they will use the “past” latent variables $s_{<i}$ as “context” when predicting the probability of a latent value $p(s_{i})$. However, the unidirectional, fixed ordering of sequence elements disregards large parts of the brain until the sequence analysis is almost complete; in order words, the transformers do not have access to the global information of the brain, and this can affects the accuracy when predicting the first elements of the sequence s. This way, anomalies will differ in their identifiability with variations in the image parts by which they are contextualised. For example, anomalies in the left hemisphere can be easier to identify if the model has access to a context where the homologous part of the right hemisphere is part of it than if it is only considering the background in the left side of the head (Fig. 4). Using different orderings, we compel each transformer to learn different interactions between parts of the image based on their availability in the model's context.

In our study, we focused on the raster scan class ordering. We obtain different orderings by reflecting the input data in different ways, e.g., for 2D experiments, we reflected the image horizontally, vertically, and both ways at the same time. We also define our orderings in inputs rotated 90 degrees, generating 8 different orderings from a single latent representation for the 2D experiments. Since the 3D experiments introduce many more combinations, we selected only 7 of them for our analysis. Each resampled latent representation is independently reconstructed, i.e., each model independently creates a residuals map. We use the mean residual to segment the anomalies.

### 2.6 Image-wise anomaly detection

So far, the proposed methodology has been focusing on segmenting abnormalities. However, transformers can also be used to perform image-wise anomaly detection, i.e., detecting if an abnormality exists somewhere in the input data. To do so, we use the likelihood predicted by the transformers. Like the segmentation approach, first, we obtain the 1D latent representation s. Then, we use the transformers to obtain the likelihood p(s) of each latent variable. To obtain the log-likelihood image-wise, we compute $logp\left(x\right)=logp\left(s\right)=\sum_{i}logp\left(s_{i}\right)$. Finally, we combined the predicted log-likelihood of each transformer (per orientation/ordering) by computing the mean value.

## 3 Experiments

### 3.1 Experiment #1 – anomaly segmentation on 2D synthetic data

First, to develop our method in a controllable scenario, where we have a large quantity of data, the delineation of the anomalies and the ability to changes its characteristics, we performed our experiments on 2D synthetic data. Training settings and model architecture are described in the supplementary materials.

**Dataset**: We utilised a subsample of the MedNIST dataset, where we used the 2D images of the “HeadCT” category to train our VQ-VAE and transformer models. From the original 10,000 HeadCT images (each one with 64×64 pixels), we used 8,000 images as the training set and 1,000 images for the validation set. The test set was comprised of 100 images contaminated with sprites (i.e., synthetic anomalies) obtained from the dsprites dataset (Matthey et al., 2017). We selected the sprites images that overlapped a significant portion of the head, and their values were set as 0 or 1.

**State-of-the-art models**: We compared our models against state-of-the-art methods (AE dense, AE spatial, f-AnoGAN and VAE). We used a network architecture adapted from a recent comparison study (Baur et al., 2020a) (more details presented in the supplementary materials).

**Results**: We measure the performance using the best achievable DICE-score (⌈DICE⌉), which constitutes a theoretical upper-bound to a model's segmentation performance and is obtained via a greedy search for the residual threshold, which yields the highest DICE-score on the test set. We also obtained the area under the precision-recall curve (AUPRC) as a sensible measure for segmentation performance under class imbalance. We compared our results against state-of-the-art autoencoder models and f-AnoGAN. We also performed an ablation study of the proposed method, demonstrating the importance and the contribution of each step.

**Fig. 5:**
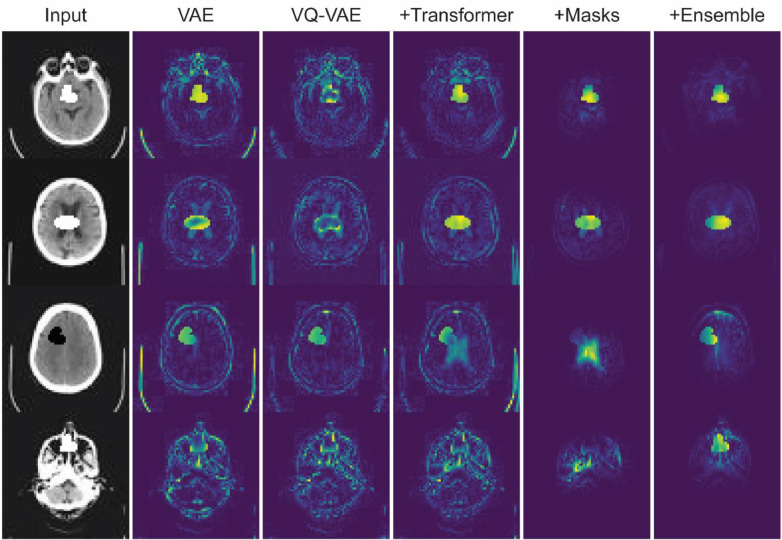
Residual maps on the synthetic examples from the variational autoencoder and different steps of our approach.

As presented in Table 1, the models without transformers exhibited a ⌈DICE⌉ no higher than 0.533 (VAE). We observed a performance improvement when using the transformer to learn latent representations distributions and resample the latent values with low probability, changing the VQ-VAE only performance from 0.457 to 0.675. The spatial information in the resampling mask also contributed by attenuating the false positives created by the blurry reconstructions (Fig. 5), achieving a 0.768 score. Finally, the variability of the autoregressive models with different orderings gave another boost in performance, achieving a ⌈DICE⌉=0.895 for eight different raster ordering models.

**Table 1:** Performance of the methods on anomaly segmentation using the synthetic dataset. The performance is measured with best achievable DICE-score (⌈DICE⌉) and area under the precision-recall curve (AUPRC) on the test set.

Method	⌈DICE⌉	AUPRC
AE (Dense) [Baur, Denner, et al., 2020]	0.213	0.129
AE (Spatial) [Baur, Denner, et al., 2020]	0.165	0.093
VAE (Dense) [Baur, Denner, et al., 2020]	0.533	0.464
f-AnoGAN [Schlegl et al., 2019]	0.492	0.432
VQ-VAE [Van Den Oord et al., 2017]	0.457	0.346
VQ-VAE + Transformer [Ours]	0.675	0.738
VQ-VAE + Transformer + Masked Residuals [Ours]	0.768	0.808
VQ-VAE + Transformer + Masked Residuals + 8 different orderings [Ours]	**0.895**	**0.956**

**Fig. 6:**
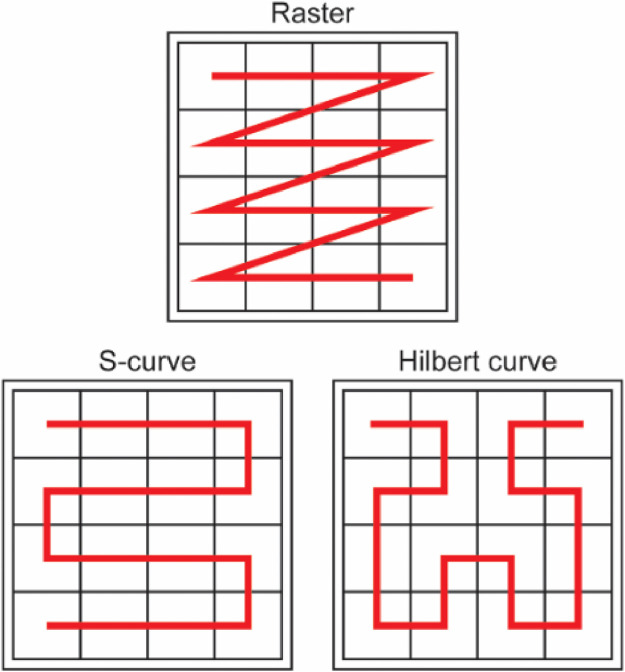
Different orderings used to transform the 2D latent representation into a 1D sequence.

**Different ordering classes**: We also analysed three other classes of orderings (Fig. 6): a S-curve order that traverses rows in alternating directions, a Hilbert space-filling curve order that generates nearby pixels in the image consecutively, and a random ordering where the sequence of latent variables was randomly sorted. Similar to the raster class, we augmented the number of possible orderings by reflecting and transposing the images, generating in total 8 different orderings per class.

In the Table 2, we can observe the performance of each ordering class. The orderings had a best achievable DICE-score varying from 0.843 to 0.895. We can observe that the random ordering achieved the lowest performance. Since the random ordering may not include the local data in the context to predict a latent value autoregressively, this might be the reason for the inferior performance as anomaly detector.

**Table 2:** Performance of our method on anomaly segmentation using different classes of ordering, and the performance when using an ensemble with all classes.

Method	⌈DICE⌉
8 different raster orderings	0.895
8 different S-curve orderings	0.883
8 different Hilbert curve orderings	0.890
8 different random orderings	0.843
32 different orderings	**0.899**

Finally, we evaluated the performance when combining all the orderings. A small gain was observed when using an ensemble of all four classes compared to the raster class only. In the following analysis, we opt to use the raster ordering to reduce the time of training and processing.

**Same ordering but different random seed**: We assume that the different ordering used in the ensemble is essential to increase the robustness of our method because different models use different parts of the input data as the context in their predictions. To verify its importance, we trained eight models using the same raster ordering but with the model parameters with different initial values (i.e., we used different random seed in each trial). We observed a drop in best achievable DICE-score when using an ensemble of transformers using the same ordering but different random seeds, from 0.895 to 0.826.

**Fig. 7:**
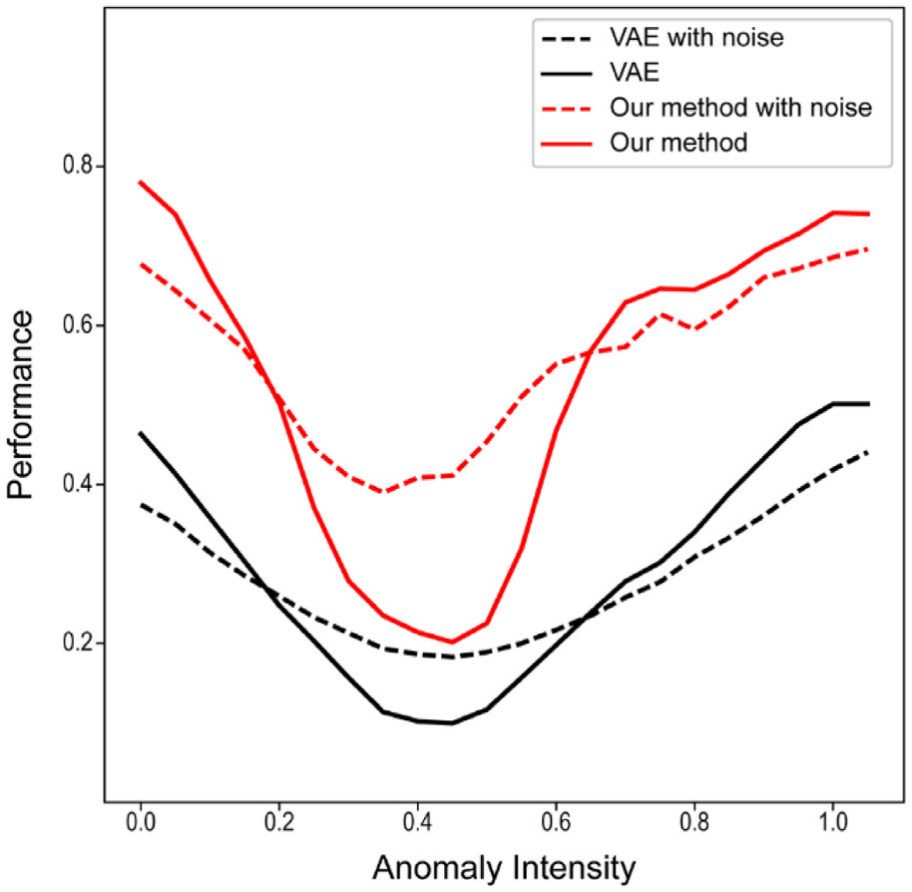
Performance with synthetic anomalies with different intensity values. We also performed the analysis including an additive Gaussian noise into the anomalies. The performance is measure by the best achievable DICE-score.

**Anomaly intensity**: We also evaluated the influence of the synthetic anomalies’ intensity and texture. For this, we varied the intensity of the sprites in the image from 0 to 1 (MedNIST images are normalized between 0-1 in our experiments) and measured the segmentation performance (best achievable DICE-score). We also performed this approach by including an additive Gaussian noise with a standard deviation of 0.2. From Fig. 7, we can observe that our transformer-based method is more robust to the change in intensity with a sharp but narrow drop in performance when the anomaly intensity is closer to the tissue mean values.

### 3.2 Experiment #2 – image-wise anomaly detection on 2D synthetic data

Next, we evaluated our method to detect anomalous (out-of-distribution - OOD) images, again in a synthetic setting.

**Dataset**: In this experiment, we used the same training set from Experiment #1. For evaluation, we used 1,000 images from the HeadCT class as the in-distribution test set, the 100 HeadCT images contaminated by sprites anomalies as the near out-of-distribution set (near OOD), and 1,000 images of each other classes from the MedNIST dataset (“AbdomenCT”, “BreastMRI”, “CXR”, “ChestCT”, and “Hand”) as the far out-of-distribution set (far OOD).

**Fig. 8:**
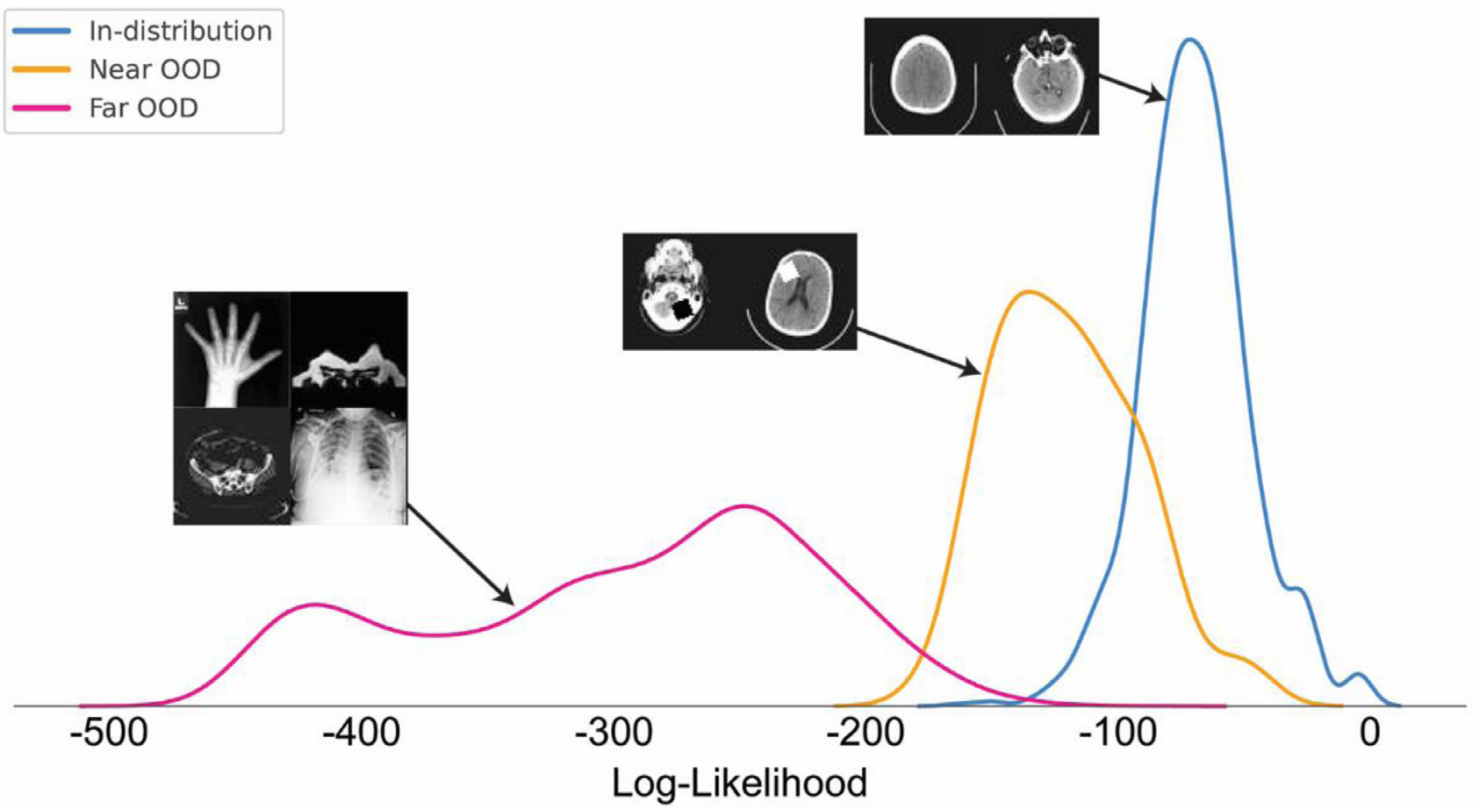
Log-likelihood distribution of the classes of examples evaluated by our ensemble of models, in-distribution, near out-of-distribution (near OOD), and far out-of-distribution (far OOD). The model assigned higher log-likelihoods for examples similar to the training set, intermediary values for examples with small synthetic lesions and lower values for examples of different classes.

**Results**: Using the log-likelihood image-wise (described in Section “Image-wise Anomaly Detection”) (Fig. 8), we use the area under the receiver operating characteristic curve (AUROC) as performance metric, with in-distribution test set and out-of-distribution being the labels. This metric permit to have a threshold-independent evaluation. We also measure the AUPRC, where it provides a meaningful measure for detection performance in the presence of heavy class-imbalance. Finally, we also computed the false positive rate of anomalous examples when the true positive rate of in-distribution examples is at 80% (FPR80), 95% (FPR95) and 99% (FPR99).

Table 3 shows that our transformer-based method achieved an AUROC of 0.921 and 1.000 for near OOD and far OOD, respectively. This is an improvement compared with a method based on the error of reconstruction obtained from a VAE model, where the reconstruction error was used to compute the performance metrics.

**Table 3:** Performance of the methods on image-wise anomaly detection using the synthetic dataset. The performance is measured using the area under the receiver operating characteristic curve (AUROC), area under the precision-recall curve (AUPRC), and false-positive rates (FPR). For the AUROC and AUPRCs, higher is better; for the FPRs, lower is better.

	AUROC	AUPRC	FPR80	FPR95	FPR99
vs. far OOD classes					
AE (Dense) [Baur, Denner, et al., 2020]	0.894	0.978	0.260	0.530	0.677
AE (Spatial) [Baur, Denner, et al., 2020]	0.785	0.953	0.506	0.845	0.881
VAE (Dense) [Baur, Denner, et al., 2020]	0.889	0.977	0.237	0.579	0.738
f-AnoGAN [Schlegl et al., 2019]	0.918	0.983	0.135	0.471	0.596
VQ-VAE [Van Den Oord et al., 2017]	0.976	0.995	0.013	0.147	0.425
Our approach	**1.000**	**1.000**	**0.000**	**0.000**	**0.001**
vs. near OOD class					
AE (Dense) [Baur, Denner, et al., 2020]	0.648	0.141	0.589	0.880	0.982
AE (Spatial) [Baur, Denner, et al., 2020]	0.663	0.142	0.526	0.838	0.926
VAE (Dense) [Baur, Denner, et al., 2020]	0.702	0.185	0.541	0.746	0.995
f-AnoGAN [Schlegl et al., 2019]	0.717	0.191	0.517	0.743	0.857
VQ-VAE [Van Den Oord et al., 2017]	0.759	0.251	0.488	0.780	0.879
Our approach	**0.921**	**0.707**	**0.102**	**0.409**	**0.885**

**General-purpose VQ-VAE for anomaly detection and segmentation**: In this analysis, we evaluated how our method performs when using a VQ-VAE trained using all classes from the MedNIST dataset. The idea was to try to mitigate the influence of the encoder in the anomaly detection tasks and just use it to compress the input data. By training the VQ-VAE with all classes, we try to reduce its ability to map an OOD image to a “healed” latent representation. If the VQ-VAE corrects the latent representation during its encoding part, the transformer will not find the anomaly area as a low likelihood area, affecting the segmentation performance.

To train our general purpose VQ-VAE, we added 8,000 images from each other classes to our training set and 1,000 images to our validation set. The ensemble of transformers was trained using only the HeadCT images. This configuration achieves slightly better performance for anomaly detection (AUROC=0.932 for near OOD and AUROC=1.000 for far OOD), and a small decrease in best achievable DICE-score for anomaly segmentation (⌈DICE⌉=0.886).

### 3.3 Experiment #3 – anomaly segmentation on real 2D neuroimaging data

Finally, we evaluate our method's performance on real-world lesion data. In this experiment, we focus on evaluating its performance using 2D slices. Training settings and model architecture are described in the supplementary materials.

**MRI Datasets**: In our experiment, we used FLAIR images from four datasets: the UK Biobank (UKB) (Sudlow et al., 2015), the White Matter Hyperintensities Segmentation Challenge dataset (WMH) (Kuijf et al., 2019), the Multimodal Brain Tumor Image Segmentation Benchmark (BRATS) (Bakas et al., 2018, 2017; Menze et al., 2014), and the Multiple Sclerosis dataset from the University Hospital of Ljubljana (MSLUB) (Lesjak et al., 2018).

The UKB is a study that aims to follow the health and well-being of 500,000 volunteer participants across the United Kingdom. From these participants, a subsample was chosen to collect multimodal imaging, including structural neuroimaging. Here, we used an early release of the project's data comprising 33,318 participants. More details about the dataset and imaging acquisition can be found elsewhere (Alfaro-Almagro et al., 2018; Elliott and Peakman, 2008; Miller et al., 2016; Sudlow et al., 2015). The UK Biobank dataset has available a mask for hyperintensities white matter lesions obtained using BIANCA (Griffanti et al., 2016; Jenkinson et al., 2012). We selected the 15,000 subjects with the lowest lesion volume to train our models (14,000 for training set and 1,000 for validation set).

The BRATS challenge is an initiative that aims to evaluate methods for the segmentation of brain tumours by providing a 3D MRI dataset with ground truth tumour segmentation annotated by expert board-certified neuroradiologists. Our study used the 2018 version of the dataset composed by the MR scans of 420 patients with glioblastoma or lower grade glioma. The images were acquired with different clinical protocols and various scanners from multiple (n = 19) institutions. Note, the available images from the BRATS dataset were already skull stripped.

The WMH dataset is an initiative to directly compare automated WMH segmentation techniques (Kuijf et al., 2019). The dataset was acquired from five different scanners from three different vendors in three different hospitals in the Netherlands and Singapore. It is composed by 60 subjects where the WMH were manually segmented according to the STandards for ReportIng Vascular changes on nEuroimaging (STRIVE) (Wardlaw et al., 2013).

The MSLUB dataset is a publicly available dataset for the validation of lesion segmentation methods. The dataset consists of 30 images from multiple sclerosis patients that were acquired using conventional MR imaging sequences. For each case, a reference lesion segmentation was created by three independent raters and merged into a consensus. This way, we have access to a precise and reliable target to evaluate segmentation methods. Full description regarding data acquisition and imaging protocol can be found at Lesjak et al. (2018).

**MRI Pre-processing**: We pre-process our images to be normalized in a common space. For this reason, all scans and lesion masks were registered to MNI space using rigid + affine transformation. This registration was performed using the Advanced Normalisations Tools (ANTs - version 2.3.4) (Avants et al., 2011). Since our anomaly segmentation method relies on a training set composed of a population with a low occurrence of lesions and anomalies, we tried to minimize the occurrence of lesions on the transformers’ training set. For this reason, after the traditional MRI pre-processing, we used the NiftySeg package (version 1.0) (Prados et al., 2016) to mitigate the influence of the lesions present in our training set. Using the *seg_FillLesions* function and the lesion maps supplied by the UKB dataset, we in-painted the few white matter hyperintensities present in the FLAIR images using a non-local lesion filling strategy based on a patch-based inpainting technique for image completion. Since the VQ-VAE performs mainly a dimensionality reduction in our method, it was trained using the normalized dataset without the NiftySeg inpainting. We believe that the presence of the lesions in the VQ-VAE training set is important to avoid the encoder performing any “healing” during the encoding process. If the encoder heals the latent code by itself, the transformer would not be able to detect the presence of a lesion. This missing detection would result in a resampling mask that filters out the encoder correction creating false negatives. In Experiment #2, we show that the presence of a lesion and anomalous classes in the VQ-VAE training set does not prejudice the performance of the segmentation. Finally, we selected four axial slices (z = 89, 90, 91, 92) per FLAIR image and, we centre cropped these slices to have the dimensions of 224×224 pixels. Before feeding the images to the models, we independently scale their values to be between 0 and 1.

**State-of-the-art Models:** We used the same unified network architecture from Baur, Denner, et al. (2020a) for the autoencoder-based and f-AnoGAN approaches (more details presented in the supplementary materials).

**Fig. 9:**
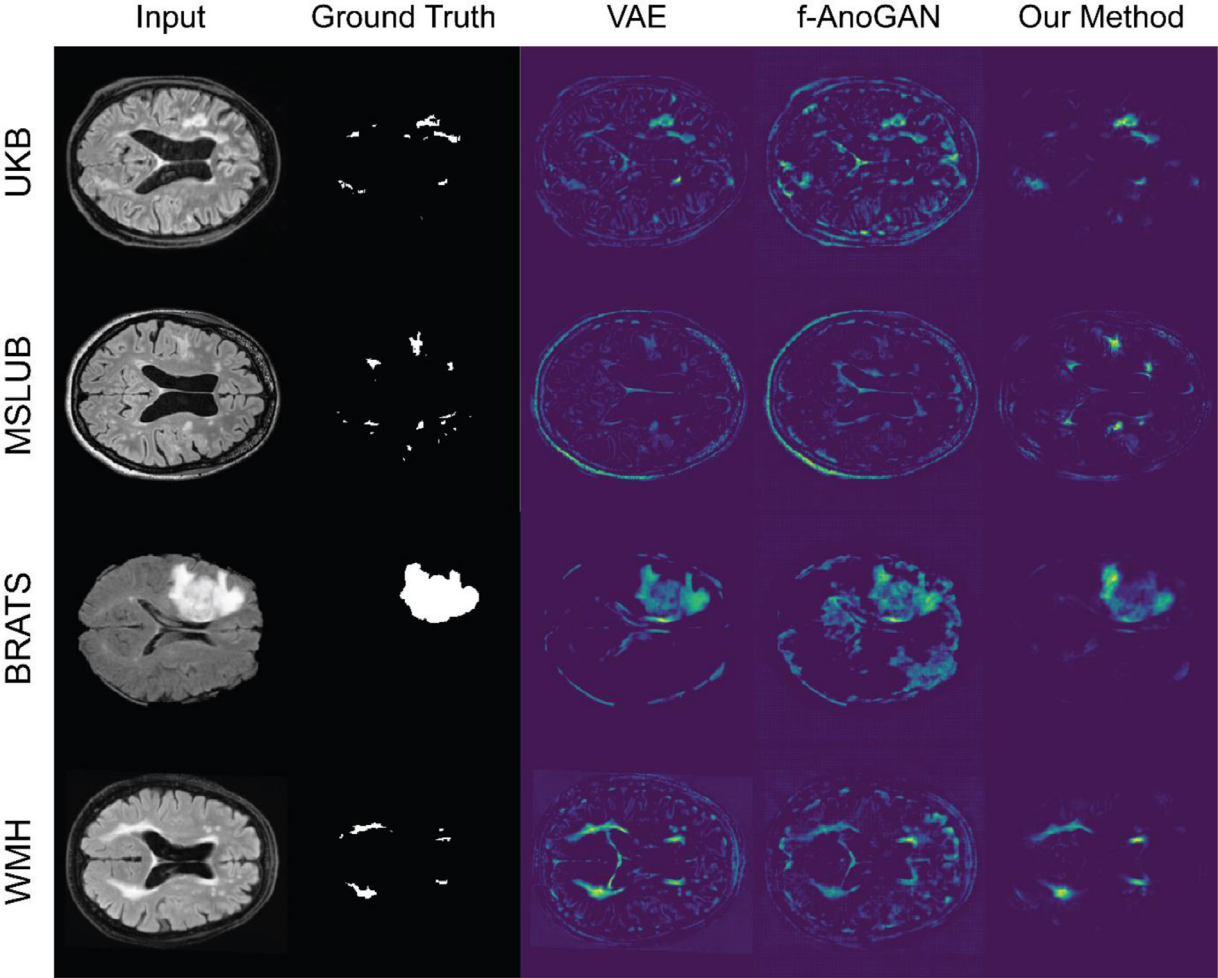
Residual maps on the real lesions from the variational autoencoder, the f-AnoGAN, and our transformer-based method.

**Results**: Our method showed a better performance than the other approaches in all datasets (Fig. 9 and Table 4). Compared to the numbers in Baur, Denner, et al. (2020a), our autoencoder-based models got a lower performance on the common dataset (MSLUB), where they achieved an best achievable DICE-score of 0.271 with the AE (dense), 0.154 with the AE (spatial), and 0.323 with the VAE (dense). We believe that the discrepancy comes mostly from their significant post-processing as presented in Table 8 of this reference. Differences might also arise from the difference in resolution, as the DICE score is not invariant to resolution.

**Table 4:** Performance on anomaly segmentation using real 2D lesion data. We compared our models against the state-of-the-art autoencoder models and f-AnoGAN model based on the architecture proposed in Baur, Denner, et al. (2020). We measured the performance using the theoretically best possible DICE-score (⌈DICE⌉) and AUPRC on each dataset.

UKB Dataset	⌈DICE⌉	AUPRC
AE (Dense) [Baur, Denner, et al., 2020]	0.016	0.005
AE (Spatial) [Baur, Denner, et al., 2020]	0.054	0.015
VAE (Dense) [Baur, Denner, et al., 2020]	0.016	0.006
f-AnoGAN [Schlegl et al., 2019]	0.060	0.025
VQ-VAE [Van Den Oord et al., 2017]	0.028	0.005
VQ-VAE + Transformer [Ours]	0.079	0.080
VQ-VAE + Transformer + Masked Residuals [Ours]	0.104	0.082
VQ-VAE + Transformer + Masked Residuals + different orderings [Ours]	**0.232**	**0.159**
**MSLUB Dataset**		
AE (Dense) [Baur, Denner, et al., 2020]	0.041	0.016
AE (Spatial) [Baur, Denner, et al., 2020]	0.061	0.026
VAE (Dense) [Baur, Denner, et al., 2020]	0.039	0.016
f-AnoGAN [Schlegl et al., 2019]	0.034	0.012
VQ-VAE [Van Den Oord et al., 2017]	0.040	0.016
VQ-VAE + Transformer [Ours]	0.097	0.050
VQ-VAE + Transformer + Masked Residuals [Ours]	0.234	0.130
VQ-VAE + Transformer + Masked Residuals + different orderings [Ours]	**0.378**	**0.272**
**BRATS Dataset**		
AE (Dense) [Baur, Denner, et al., 2020]	0.159	0.094
AE (Spatial) [Baur, Denner, et al., 2020]	0.329	0.215
VAE (Dense) [Baur, Denner, et al., 2020]	0.173	0.107
f-AnoGAN [Schlegl et al., 2019]	0.243	0.167
VQ-VAE [Van Den Oord et al., 2017]	0.197	0.125
VQ-VAE + Transformer [Ours]	0.288	0.226
VQ-VAE + Transformer + Masked Residuals [Ours]	0.328	0.292
VQ-VAE + Transformer + Masked Residuals + different orderings [Ours]	**0.537**	**0.555**
**WMH Dataset**		
AE (Dense) [Baur, Denner, et al., 2020]	0.073	0.024
AE (Spatial) [Baur, Denner, et al., 2020]	0.150	0.054
VAE (Dense) [Baur, Denner, et al., 2020]	0.068	0.022
f-AnoGAN [Schlegl et al., 2019]	0.048	0.017
VQ-VAE [Van Den Oord et al., 2017]	0.100	0.036
VQ-VAE + Transformer [Ours]	0.205	0.109
VQ-VAE + Transformer + Masked Residuals [Ours]	0.269	0.158
VQ-VAE + Transformer + Masked Residuals + different orderings [Ours]	**0.429**	**0.320**

**Post-processing Impact**: Similar to Baur et al. (2020), we verified the performance of the methods using the prior knowledge that multiple sclerosis lesions would appear as positive residuals as these lesions appear as hyper-intense in FLAIR images. We assumed the same for the white matter hyperintensities. Using only the residuals' positive values as a post-processing step, we observed an improvement in the autoencoders-based methods, the f-AnoGAN method, and our approach (Table 5).

**Table 5:** Performance on anomaly segmentation using post-processing step.

UKB Dataset	⌈DICE⌉
AE (Dense) + post-processing [Baur, Denner, et al., 2020]	0.079
AE (Spatial) + post-processing [Baur, Denner, et al., 2020]	0.054
VAE (Dense) + post-processing [Baur, Denner, et al., 2020]	0.071
f-AnoGAN + post-processing [Schlegl et al., 2019]	0.112
VQ-VAE + post-processing [Van Den Oord et al., 2017]	0.046
VQ-VAE + Transformer + Masked Residuals + different orderings + post-processing [Ours]	**0.297**
**MSLUB Dataset**	
AE (Dense) + post-processing [Baur, Denner, et al., 2020]	0.106
AE (Spatial) + post-processing [Baur, Denner, et al., 2020]	0.067
VAE (Dense) + post-processing [Baur, Denner, et al., 2020]	0.106
f-AnoGAN + post-processing [Schlegl et al., 2019]	0.057
VQ-VAE+ post-processing [Van Den Oord et al., 2017]	0.077
VQ-VAE + Transformer + Masked Residuals + different orderings + post-processing [Ours]	**0.465**
**WMH Dataset**	
AE (Dense) + post-processing [Baur, Denner, et al., 2020]	0.166
AE (Spatial) + post-processing [Baur, Denner, et al., 2020]	0.151
VAE (Dense) + post-processing [Baur, Denner, et al., 2020]	0.161
f-AnoGAN + post-processing [Schlegl et al., 2019]	0.110
VQ-VAE + post-processing [Van Den Oord et al., 2017]	0.143
VQ-VAE + Transformer + Masked Residuals + different orderings + post-processing [Ours]	**0.441**

**Impact of Mitigating Lesions in the Training set**: In our pre-processing, we in-painted the white matter hyperintensity of the training set using the NiftySeg package to simulate completely lesion-free data. Our method without this step exhibited a drop in the best achievable DICE-score, from 0.232 to 0.051 in the UKB dataset, from 0.378 to 0.264 in the MSLUB dataset, from 0.429 to 0.349 in the WMH dataset, and from 0.759 to 0.677 in the BRATS dataset. We believe that the highly expressive transformers can learn from the few white matter hyperintensities present in the original dataset and associate a higher probability of occurrence, decreasing detection performance.

### 3.4 Experiment #4 – anomaly segmentation on real 3D neuroimaging data

Three-dimensional imaging is widely used in research and clinical practice, and it allows us to obtain essential information about the global condition of the brain. Since brain lesions and pathological deviations have a 3D structure, the ability of an anomaly detector to explore the third dimension is crucial for its success. However, the use of 3D deep neural networks is challenging in nature as it results in increased computational requirements. In this experiment, we evaluate our segmentation method's performance on 3D real-world lesion data. Training settings and model architecture are described in the supplementary materials.

**Dataset and MRI Pre-processing**: We used the high-resolution volumes from the pre-processed FLAIR images from Experiment #3, where each volume has 192×224×192 voxels. We use the same training set from the UKB for our models, where we use the data version corrected by NiftySeg to train our transformers. In contrast with the previous experiment, we use a percentile scaling (using percentile 1 and 99) to scale the values of the volumes to be between 0 and 1.

**Results**: Our method showed a better performance than the variational autoencoder approach in all datasets (Table 6). Compared with the results from Experiment #3 (2D data), we observed that our method had higher performance on the UKB data (the same dataset from which we extracted the training set) while presenting a lower performance in the other datasets. As reported in previous studies, 3D models are more difficult to generalise well. For example, in Bengs et al. (2021), the authors rely on several regularisation strategies to make their unsupervised anomaly detectors achieve higher DICE scores with a 3D architecture compared to their 2D architecture.

**Table 6:** Performance of the methods on anomaly segmentation using real 3D lesion data. We measured the performance using the theoretically best possible DICE-score (⌈DICE⌉) on each dataset.

UKB Dataset	⌈DICE⌉
VAE (Dense) [Baur, Denner, et al., 2020]	0.018
VQ-VAE + Transformer + Masked Residuals + different orderings [Ours]	**0.368**
**MSLUB Dataset**	
VAE (Dense) [Baur, Denner, et al., 2020]	0.021
VQ-VAE + Transformer + Masked Residuals + different orderings [Ours]	**0.133**
**BRATS Dataset**	
VAE (Dense) [Baur, Denner, et al., 2020]	0.192
VQ-VAE + Transformer + Masked Residuals + different orderings [Ours]	**0.617**
**WMH Dataset**	
VAE (Dense) [Baur, Denner, et al., 2020]	0.021
VQ-VAE + Transformer + Masked Residuals + different orderings [Ours]	**0.133**

### 3.5 Experiment #5 – image-wise anomaly detection on real 3D neuroimaging data

In this last experiment, we evaluated our method to identify subjects with a diagnosis on their hospital inpatient records from subjects with no reported diagnosis in their records.

**Dataset**: In this experiment, we use only the subjects from the UKB. We used the diagnosis codes that each UKB participant has recorded across all their hospital inpatient records (fields 41202-0 and 41204-0 “Diagnoses – main and secondary ICD 10”) to select the subjects. From the test set from Experiment #4, we selected the subjects that had the diagnosis for multiple sclerosis (diagnosis code “G35”), resulting in 60 participants. As “healthy control” group, we created a balanced group (for age and gender) by selecting 60 subjects from the test set that did not have any inpatient record and had a lesion size smaller than 5000 voxels according to the UKB lesion masks. In this experiment, we verify the performance of our models to detect anomalies using only the likelihood obtain from the transformers (similar to Experiment #2), and we also use the lesion size predicted by our segmentation algorithm. We used the residual maps from the validation set (1000 subjects) to determine the best threshold to apply to the residual mask and create binary masks. Since we include two variables to perform anomaly detection, we are also training a one-class support vector machine (OC-SVM) on the validation set.

**Fig. 10:**
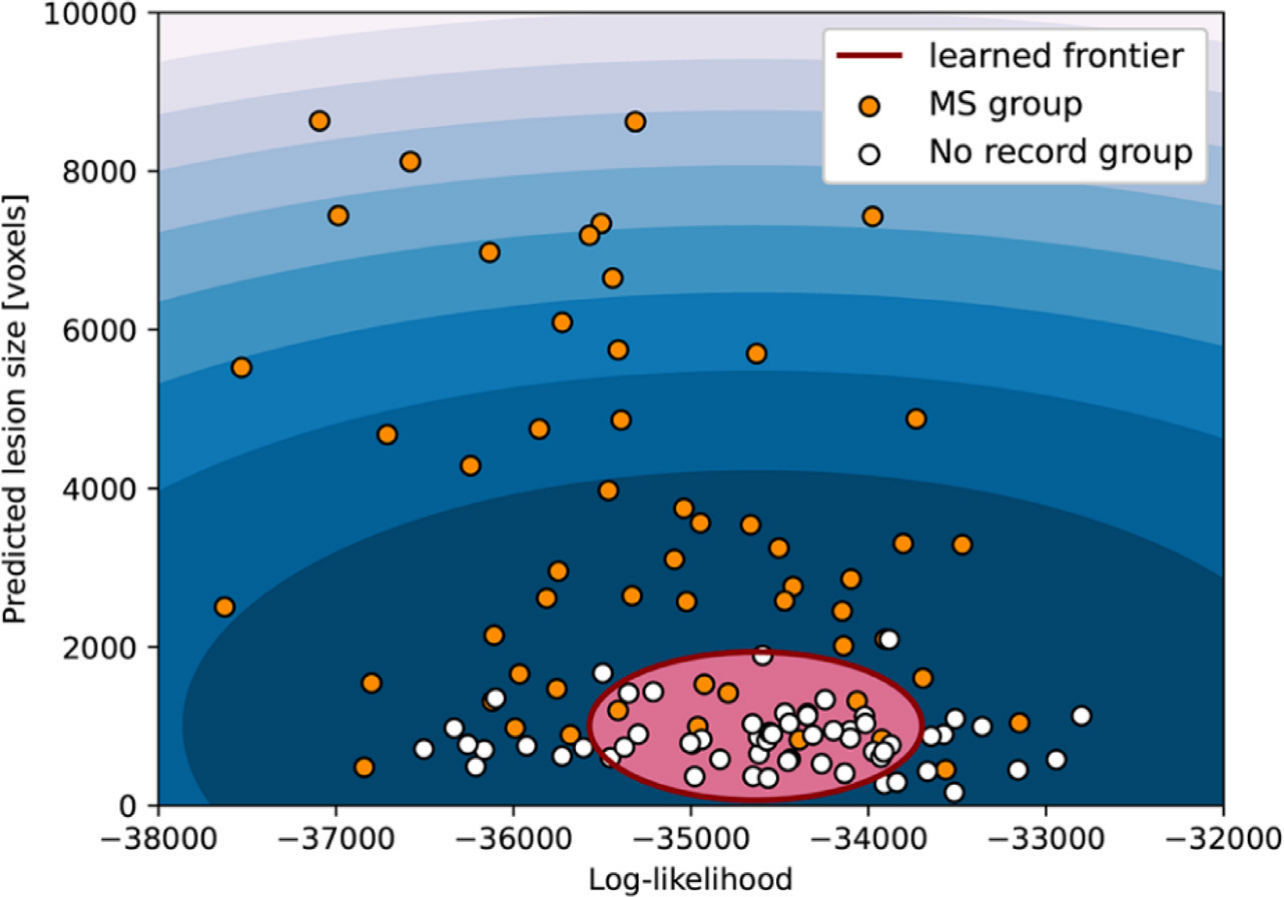
Anomaly detection image-wise on 3D data. In this experiment, we use the log-likelihood obtained from the transformers and the lesion size from the binary mask predicted by our models to train a one-class SVM and classify subjects with multiple sclerosis diagnosis in their records as out of distribution.

**Results**: Using only the log-likelihood image-wise from the transformers, our method achieved an AUCROC of 0.698 when classifying the subjects with multiple sclerosis diagnosis in their hospital inpatient records from subjects with no reported diagnosis code. Our performance increase to an AUCROC of 0.866 when we include the lesion segmentation component (Fig. 10).

## 4 Conclusion

Automatically determining the presence of lesions and delineating their boundaries is essential to the introduction of complex models of rich neuroimaging features in clinical care. In this study, we propose a novel transformer-based approach for anomaly detection and segmentation that achieves state-of-the-art results in all tested tasks when compared with competing methods. Transformers are making impressive gains in image analysis, and here we show that their use to identify anomalies holds great promise. We hope that our work will inspire further investigation of the properties of transformers for anomaly detection in medical images, the development of new network designs, exploration of a wider variety of conditioning information, and the application of transformers to other medical data.

## CRediT authorship contribution statement

**Walter H.L. Pinaya:** Conceptualization, Methodology, Software, Investigation, Writing – original draft. **Petru-Daniel Tudosiu:** Conceptualization, Methodology, Writing – review & editing. **Robert Gray:** Conceptualization, Methodology, Writing – review & editing. **Geraint Rees:** Writing – review & editing, Funding acquisition. **Parashkev Nachev:** Conceptualization, Methodology, Supervision, Writing – review & editing, Funding acquisition. **Sebastien Ourselin:** Writing – review & editing, Funding acquisition. **M. Jorge Cardoso:** Conceptualization, Methodology, Supervision, Writing – review & editing, Funding acquisition.

## Declaration of Competing Interest

The authors declare that they have no known competing financial interests or personal relationships that could have appeared to influence the work reported in this paper.

## Appendix Supplementary materials

Image, application 1
