# Supplementary material for: Unsupervised brain imaging 3D anomaly detection and segmentation with transformers
Source: Med Image Anal. 2022 Jul;79:102475. doi: 10.1016/j.media.2022.102475 (PMC10108352; doi:10.1016/j.media.2022.102475)
Supplement: Supplementary file 1 [file mmc1.docx]

**Unsupervised Brain Imaging 3D Anomaly Detection and Segmentation with Transformers**

Walter H. L. Pinaya ^1, *^, Petru-Daniel Tudosiu ^1^, Robert Gray ^2^, Geraint Rees ^3^, Parashkev Nachev ^2^, Sebastien Ourselin ^1^, M. Jorge Cardoso ^1^

^1^ Department of Biomedical Engineering, School of Biomedical Engineering & Imaging Sciences, King's College London, London, UK

^2^ UCL Queen Square Institute of Neurology, University College London, London, UK

^3^ UCL Faculty of Life Sciences, University College London, London, UK

# Experiments – Supplementary details

## Experiment #1 – Anomaly Segmentation on 2D Synthetic Data

**Models**: Our VQ-VAE models had a similar architecture from Van Den Oord et al. (2017). The encoder consists of three strided convolutional layers with stride 2 and window size 4 × 4. All these convolution layers had a ReLU activation following them. This structure is followed by two residual 3×3 blocks (implemented as 3×3 conv, ReLU, 1×1 conv, ReLU). Similarly, the decoder has two residual 3×3 blocks, followed by three transposed convolutions with stride 2 and window size 4×4. All the convolution layers have 256 hidden units. The inputted images have 64x64 pixels which result in a latent representation of 8x8 latent variables. For this experiment, we arbitrary choose a codebook with 16 different codes. Our performers corresponded to the transformer’s decoder structure with 24 layers with an embedding size of 256. The embedding, feed-forward and attention dropout had a probability of 0.1.

**Training settings**: To train the VQ-VAE models, we use the ADAM optimiser with a learning rate 5e-4, an exponential learning rate decay with a gamma of 0.999, and we trained over 1,500 epochs with batch-size 256. We train the codebook using an exponential moving average algorithm. To stabilise the codebook’s learning, in the first 100 epochs, we warm up the moving average decay from a gamma decay of 0.5, gradually increasing to a gamma decay of 0.99. The warm-up allows the codes to adapt faster to the frequent changes at the beginning of the training. To train the performers, we used the ADAM optimiser with a learning rate 5e-4, an exponential learning rate decay with a gamma of 0.9999, and we trained over 1,500 epochs with batch-size 128. We used data augmentation to increase the number of training images. We randomly performed affine transformations (scale, translate, and rotate operations) and horizontally flipped the images.

The training was performed in an NVIDIA DGX-2 server within a Docker container with access 1 GPU TESLA V100-SXM3-32GB, and it took 8 hours to train the VQVAE and 28 hours to train each transformer. Each VQ-VAE model had 7M parameters, and each transformer had 20M parameters.

**State-of-the-art models**: We compared our models against state-of-the-art autoencoder-based methods (AE dense, AE spatial, and VAE) and f-AnoGAN. We used a network architecture adapted from a recent comparison study (Baur et al., 2020). Since MedNIST images are smaller than those used in the study, our models did not have the first block (resolution of 64x64x32) and the last block (resolution of 128x128x32). All the other blocks and layers are similar to the original study. To train these models, we used the ADAM optimiser with a learning rate 5e-4, an exponential learning rate decay with a gamma of 0.9999, and we trained over 1500 epochs with batch-size 256. For f-AnoGAN, since the training of the discriminator is unstable (e.g., presenting mode collapse), in its first stage (i.e., the adversarial training), we used the checkpoint right before the networks collapsed.

## Experiment #2 – Image-wise Anomaly Detection on 2D Synthetic Data

**Models and Training Settings**: We used the same trained models from Experiment #1.

## Experiment #3 – Anomaly Segmentation on Real 2D Neuroimaging Data

**Models**: Our VQ-VAE models had a similar architecture from Experiment #1 but with three residual 3×3 blocks (instead of two). All the convolution layers had 256 hidden units. The inputted images had 224x224 pixels which result in a latent representation of 28x28 latent variables. We arbitrary choose a codebook with 32 different codes. Our performers had 16 layers with an embedding size of 256, using a dropout with probability of 0.3.

**Training settings**: To train the VQ-VAE models, we use the ADAM optimiser with learning rate 1e-3, an exponential learning rate decay with a gamma of 0.99995, and we trained over 500 epochs with batch-size 256. We train the codebook similar to Experiment #1. To train the performers, we used the ADAM optimiser with a learning rate 1e-3, an exponential learning rate decay with a gamma of 0.99992, and we trained over 150 epochs with batch-size 128. We used data augmentation to increase the number of training images. We randomly performed small translation transformations as well as random intensity shift and random adjusts in contrast.

The training was performed in an NVIDIA DGX-2 server within a Docker container with access 4 GPU TESLA V100-SXM3-32GB, and it took 12 hours to train the VQVAE and 22 hours to train each transformer. Each VQ-VAE model had 8M parameters, and each transformer had 13M parameters.

**State-of-the-art Models:** We used the same unified network architecture from Baur, Denner, et al. (2020) for the autoencoder-based approaches and f-AnoGAN. To train these models, we used the ADAM optimiser with a learning rate 1e-3, an exponential learning rate decay with a gamma of 0.99995, and we trained over 1500 epochs with batch-size 256. In this experiment, we also had instability problems with f-AnoGAN and used the same approach from Experiment #1.

## Experiment #4 – Anomaly Segmentation on Real 3D Neuroimaging Data

**Models**: Our VQ-VAE model is slightly different from the other experiments. The encoder consists of two strided convolutional layers with 128 hidden units, stride 2, and window size 4 × 4. All these convolution layers had a ReLU activation following them. This structure is followed by three residual 3×3 blocks with 128 hidden units, a strided convolutional layer, and more three residual 3×3 blocks with 256 hidden units. The decoder has a symmetric architecture. The inputted volumes resulted in a latent representation of 24x28x24 latent variables. For this experiment, we arbitrary choose a codebook with 64 different codes. Our performers had 20 layers with an embedding size of 256.

**Training settings**: To train the VQ-VAE model, we use the ADAM optimiser with a learning rate 3e-4, an exponential learning rate decay with a gamma of 0.9999, and we trained over 200 epochs with batch-size 32. We train the codebook similar to Experiment #1. To train the performers, we used the ADAM optimiser with a learning rate 2e-3, an exponential learning rate decay with a gamma of 0.99997, and we trained over 30 epochs with batch-size 4.

The training was performed in an NVIDIA DGX-2 server within a Docker container with access 4 GPU TESLA V100-SXM3-32GB, and it took 42 hours to train the VQVAE and 107 hours to train each transformer. Each VQ-VAE model had 20M parameters, and each transformer had 25M parameters.

**State-of-the-art Models:** We used a similar architecture to Experiment #3 but with an extra downsampling step due to the limitation in computational resources. To train these models, we used the ADAM optimiser with a learning rate 1e-3, an exponential learning rate decay with a gamma of 0.9999, and we trained over 200 epochs with batch-size 16.

## Experiment #5 – Image-wise Anomaly Detection on Real 3D Neuroimaging Data

**Models and Training Settings**: We used the same VQ-VAE and transformers trained in Experiment #4. For our OC-SVM, we are using the radial basis function kernel.

# Ablation studies

## Size of codebook

In this ablation study, we compute the influence of the number of codes in the anomaly segmentation test using the Experiment #1 and #3 scenarios. Here, we evaluated the performance of the VQ-VAE + Transformer. The results are presented in Table S1 and S2. For Experiment #1, we observed that the number of codes had a peak performance at 128 codes, while Experiment #3 had a peak performance at 16 and 64 codes. Scenarios with more codes present a decrease in performance, and we believe that this is due to the complexity of latent representations. Since the transformer structure is the same across the evaluated scenarios, a more complex representation (with more distinct codes) might not be learned well due to the low capacity of the models.

Table S1 – Influence of the number of codes in the anomaly segmentation method on Experiment #1. The performance is measured with best achievable DICE-score (⌈DICE⌉).

| **Number of Codes** | **⌈DICE⌉** |
| --- | --- |
| 8 | 0.635 |
| 16 | 0.675 |
| 32 | 0.705 |
| 64 | 0.682 |
| **128** | **0.713** |
| 256 | 0.648 |
| 512 | 0.637 |
| 1024 | 0.623 |

Table S2 – Influence of the number of codes in the anomaly segmentation method on Experiment #3. The performance is measured with best achievable DICE-score (⌈DICE⌉) o the MSLUB dataset.

| Number of Codes | MSLUB ⌈DICE⌉ |
| --- | --- |
| 8 | 0.226 |
| 16 | **0.246** |
| 32 | 0.234 |
| 64 | **0.246** |
| 128 | 0.203 |
| 256 | 0.150 |
| 512 | 0.128 |
| 1024 | 0.101 |

# Experiment #3 examples


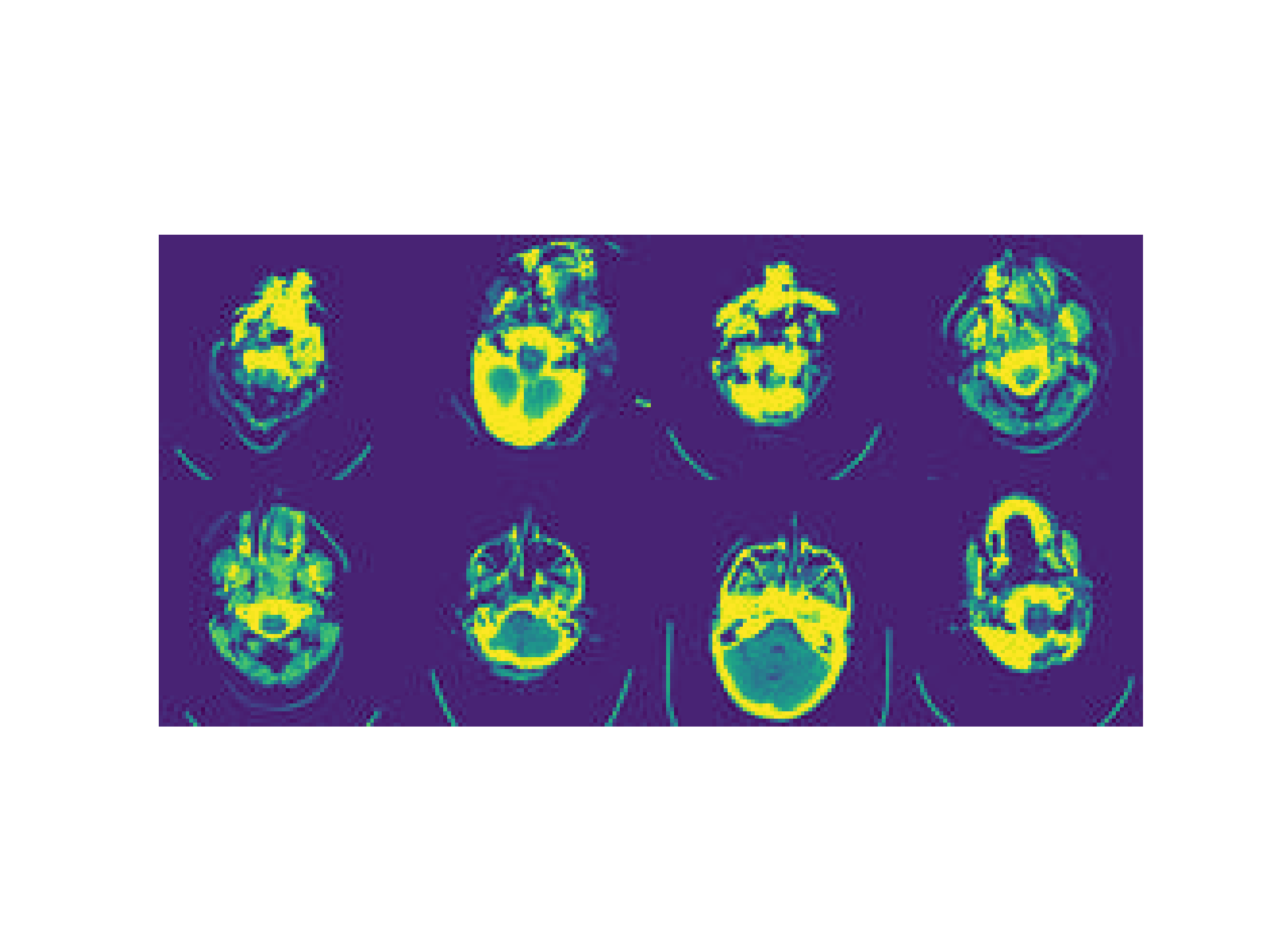


Figure S1 – Examples from the in-distribution test set that had the lowest likelihood according to our ensemble of transformers.


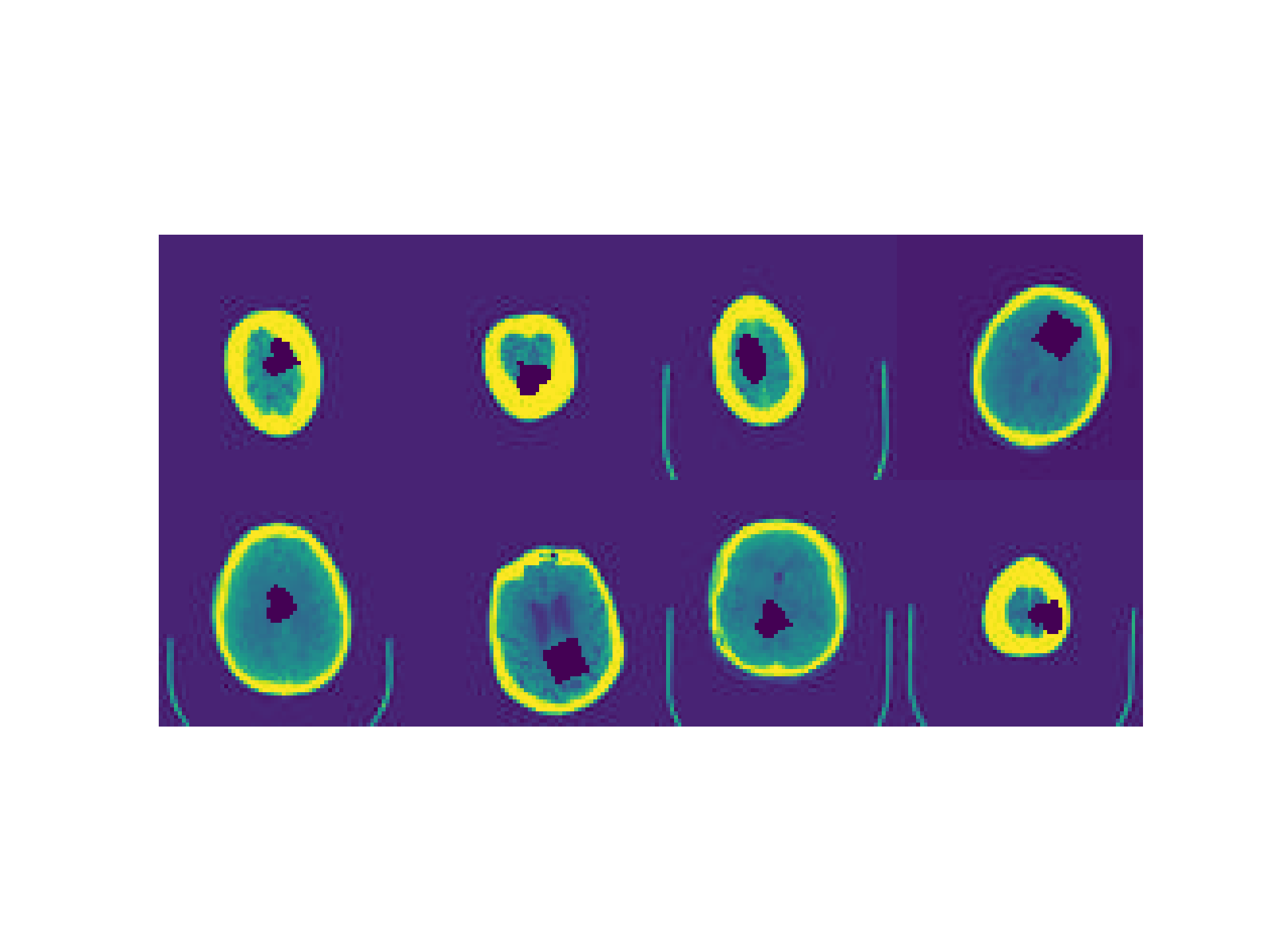


Figure S2 – Examples from the near out-of-distribution set that had the highest likelihood according to our ensemble of transformers.


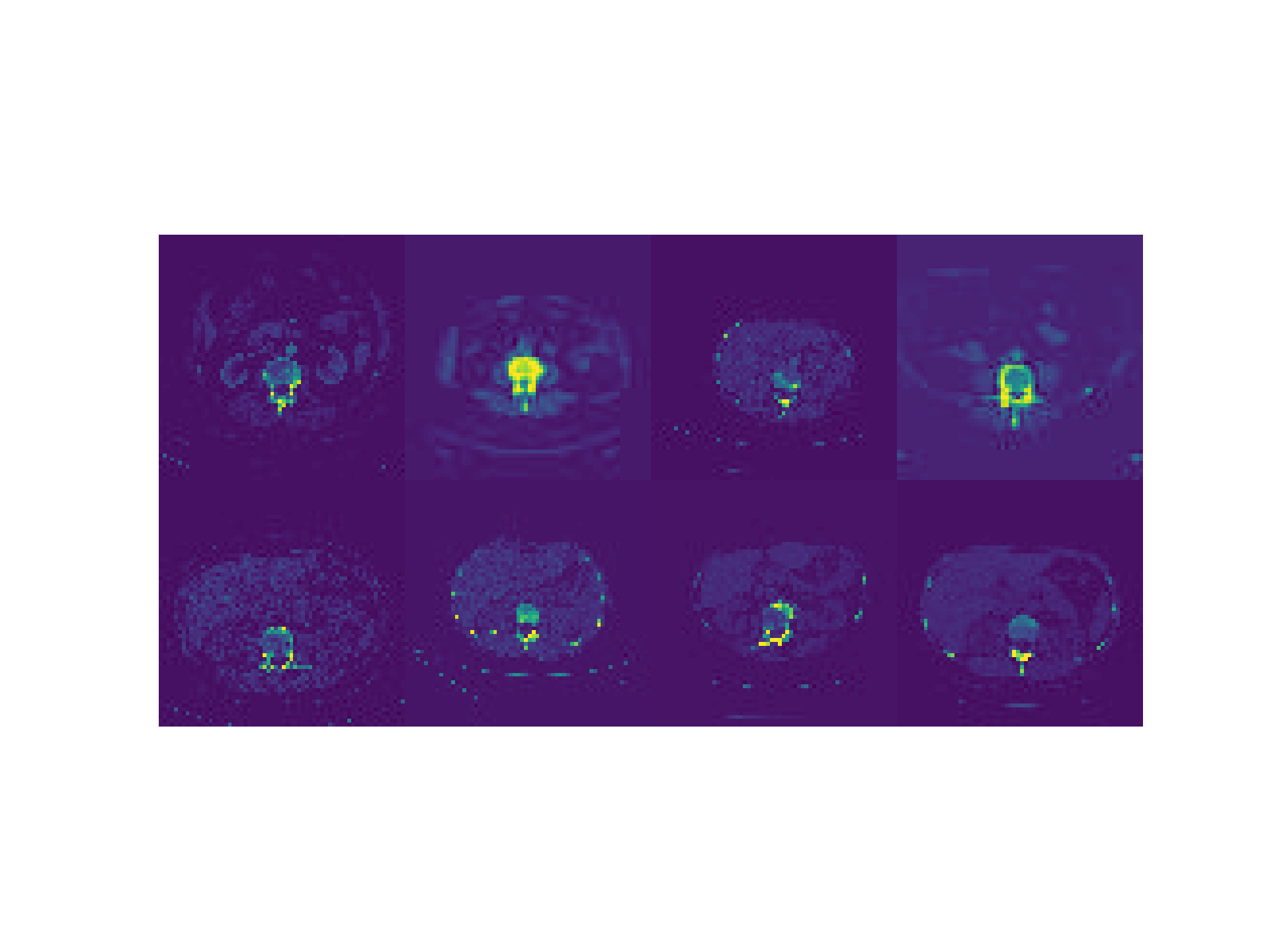


Figure S3 - Examples from the far out-of-distribution set that had the highest likelihood according to our ensemble of transformers.

# References

Baur, C., Denner, S., Wiestler, B., Albarqouni, S., Navab, N., 2020. Autoencoders for Unsupervised Anomaly Segmentation in Brain MR Images: A Comparative Study. arXiv Prepr. arXiv2004.03271.

Van Den Oord, A., Vinyals, O., Kavukcuoglu, K., 2017. Neural discrete representation learning. Adv. Neural Inf. Process. Syst. 2017-Decem, 6307–6316.
